# Influence of Formulation on Mobility of Clomazone in Soil

**DOI:** 10.1007/s00128-016-1903-7

**Published:** 2016-08-24

**Authors:** Małgorzata Włodarczyk, Hanna Siwek

**Affiliations:** Faculty of Environmental Management and Agriculture, West Pomeranian University of Technology in Szczecin, ul. Słowackiego 17, 71-176 Szczecin, Poland

**Keywords:** Clomazone, Formulation, Mobility, Soil, Alginate, Column test

## Abstract

The mobility of clomazone [2-(2-chlorobenzylo)-4,4-dimetylo-1,2-oxazolidin-3-one] in a loamy sand soil and a sand soil was studied in a soil column under laboratory conditions. Commercial clomazone formulation (Command 480 EC) and clomazone immobilized in an alginate matrix were used for a leaching experiment. For both formulations, the same dose of 2.0 mg of the active substance was applied. After an application of a herbicide, the columns were irrigated with: 100, 40 and 3.7 mm of water. After 1 h, when an addition of water was completed, the soils were sampled in the 5 cm segments and were used for the analysis of the residues. The use of an alginate formulation reduced the vertical mobility of clomazone into a soil layer in comparison with the formulation EC.

Pesticides constitute a significant element of modern agriculture and are mainly used for destroying weeds, controlling pests and fighting plant diseases. However, due to the wide-spread use of pesticides, there has been a great concern about their toxicity towards non-target organisms and the natural environment (Van der Werf 1996; Alister and Kogan 2006; Arias-Estevez et al. 2008). There are various factors which affect pesticide activity in the environment. They can be directly connected with the agricultural production (such as a time, a type and a mode of the application, a dose), the environmental factors (the atmospheric conditions, a terrain, a type of a soil), the physiochemical properties of an active substance, or a pesticide formulation. Pesticide activity is regulated by a variety of complex, dynamic, physical, chemical and biological processes including sorption – desorption, evaporation, chemical and biological degradation, plant uptake, surface runoff or leaching. These processes determine pesticide mobility in a soil, as well as translocation from a soil to water, air or food (Reddy 1993; El-Nahhal 2003; Sondhia 2009; Urlich et al. 2013). To reduce pesticide pollution, the efforts have been made to improve agricultural pest management, such as developing new biodegradable pesticides, producing the new types of formulations (concentrated in a suspension, granules, the soluble liquids, the ionic liquids) (Mulqueen 2003; Green and Beestman 2007; Knowles 2008; Pernak et al. 2011) as well as formulations based on controlled release (CR) technology (Mogul et al. 1996; Sopeña et al. 2008). CR formulations can regulate or reduce the rate of availability of a pesticide, localizing the chemicals in the crop zone and reducing the amount accessible to leaching processes. The parameters that affect the properties of CR formulation depend on the nature and type of polymer used (Cao et al. 2005; Maqueda et al. 2009; Sopeña et al. 2009; Roy et al. 2014). Several research studies have contributed to the development of formulations for the controlled release in agriculture. Cotterill et al. (1996) found that lignins from the different sources can be used for the matrix formulations, resulting in varying the release rate. The release of pesticides from the lignin matrix depends on the type of the lignin, methods of bonding pesticides in the lignin matrix and the granule size. The CR formulations of isoproturon, imidacloprid and cyromazine have been evaluated by Fernández-Pérez et al. (2011).The effects of two natural polymers (alginate and lignin) and two modifying sorbents (bentonite and activated carbon) on the pesticide release kinetics from the CR formulations have been investigated, as well as the mobility of pesticides using the soil columns. The rate of the pesticide release in the soil from the CRFs diminished in all the cases in relation to the technical products. Fernández-Pérez et al. (2000a, b, 2001) showed that the alginate-bentonite CR formulations might constitute the efficient system for reducing atrazine, isoproturon and carbofuran leaching in the layered soil. Moreover, the laboratory tests showed that the modifying agents like anthracite, bentonite, active carbon of the alginate formulations reduce the release rate of chloridazon, metribuzin in comparison with the technical products, and with the alginate formulations without modifying agents (Flores-Céspedes et al. 2007). Pepperman and Kuan (1993, 1995) found that the use of linseed oil and alginate as the basis of the CR formulation of alachlor and metribuzin results in reducing the release of the herbicide in comparison with the conventional formulation. The development of CR formulations of alachlor and atrazine based on ethylcellulose increased their herbicidal activity and reduced the loss by leaching in the soil. In the case of atrazine incorporated in ethylcellulose, matrix modifiers (allophanic clays and nanoclays) had a slight effect on the atrazine release into water, its dissipation and its behavior in the soil compared with the formulation without them (Sopeña et al. 2007; Cea et al. 2010).

The herbicide clomazone [2-(2-chlorobenzylo)-4,4-dimetylo-1,2-oxazolidin-3-one] is an isoxazolidinone group of a broad spectrum, a pre-emergence selective herbicide with both a contact and a residual activity which controls the annual broad leaf weeds and grasses. Clomazone is highly water-soluble, minimally volatile, resistant to a hydrolysis under a wide range of pH values. Clomazone is weakly sorptive to a soil, and its sorption depends on a carbon content. The field dissipation half-life of clomazone, determined from the field studies in the several soil types, ranged from 4 to 16 weeks (Mervosh et al. 1995; Antonious 2000; Lian-Fang et al. 2004; Bhattacharyya et al. 2014). Due to these characteristics, clomazone is persistent in water environment and causes a groundwater contamination. Clomazone residues were detected in the water samples collected from the wells (50 %–70 %) and the rivers (90 %) in the rice cultivation region (Zanella et al. 2002; Caldas et al. 2010).

The aim of the present study was to determine the influence of the formulation on the mobility of clomazone in the soil.

## Materials and Methods

Two formulations of herbicide clomazone were used in the experiment: emulsifiable concentrate (EC) in the form of a commercial preparation Command 480 EC, and in the form of the capsules based on the alginate matrix. The alginate capsules of clomazone were obtained in the Center of Bioimmobilization and Innovative Packaging Materials at the West Pomeranian University of Technology in Szczecin, Poland (Włodarczyk et al. 2010). The active substance with the purity of 99.9 %, was provided by Chemical Plant “Organika-Sarzyna”, Poland. Selected physical and chemical properties of clomazone are presented in Table 1.

**Table 1:** Selected physical and chemical characteristics of clomazone. http://sitem.herts.ac.uk/aeru/ppdb/en/Reports/168.htm

Structure diagram	IUPAC Name	2-(2-chlorobenzylo)-4,4-dimetylo-1,2-oxazolidin-3-one
	Physical state	White crystalline solid
	Chemical formula	C_12_H_14_ClNO_2_
	Molecular mass	239.7 g·mol^−1^
	Solubility in water	1102 mg·L^−1^ (20°C)
	Octanol/water partition coefficient:	log K_ow_ = 2.54

Two soils were used in this experiment. Both soils were taken from Western Pomerania Region, Poland. Soil samples were selected according to their content of the organic carbon and collected at a 0–20 cm depth, air dried, homogenized and passed through a 2.0 mm sieve. The physicochemical and granulometrical characteristics of these soils are given in Table 2.

**Table 2:** Selected properties of soils

Soil	Granulometric group	Water capacity	Organic carbon	pH		Hh	CEC	TEB	BS
		(%)	(%)	H_2_O	KCl	(cmol·kg^−1^)			(%)
S1	Loamy sand soil	34.63	0.83	5.56	4.28	3.33	16.10	19.43	82.88
S2	Sand soil	37.18	1.99	4.56	3.71	10.33	15.30	25.63	59.71

*Hh* hydrolytic acidity, *CEC* cation exchange capacity, *TEB* total exchangeable bases, *BS* base saturation

Mobility of clomazone in the soil was tested using PCV columns (ca. 40 cm long, diameter Φ = 3.0 cm) filled with the air-dry soil. The height of the soil stack in the column was 20 cm, the spraying area was 0.0007 m^2^. For both formulations, the same dose of 2.0 mg of the active substance was applied. Both herbicide treatments (solution of Command 480 EC 2.0 mg·mL^−1^ and alginate capsules 0.056 g) were applied to triplicate the soil columns. Then, the columns were irrigated with a dose of water corresponding to the amount of a rainfall characteristic for Western Pomeranian Region, Poland: 100 mm (a maximum rainfall), 40 mm (an average maximum rainfall) and 3.7 mm (an average rainfall) (Koźmiński et al. 2012). Water was dispensed by a peristaltic pomp with a flow rate of 0.05 mL·min^−1^. Having completed the application of water (24 h), after one extra hour (to minimize the degradation process) the content of the column was divided into the 5-centimeter pieces, for which the active substance concentration were determined (0–5 cm layer I, 5–10 cm layer II, 10–15 cm layer III, 15–20 cm layer IV). In the case of the maximum rainfall, the water elutes (layer V) collected during the experiment were made a subject to an extraction too.

The wet soil samples were extracted with 75 mL of acetone. The samples were shaken for 4 h in a mechanical shaker and then filtered. Next, acetone was evaporated in a rotary vacuum evaporator (35°C) and the residues (water samples) were liquid–liquid extracted with chloroform. Extracts were dehydrated with anhydrous sodium sulfate, purified on the columns filled with florisil and anhydrous Na_2_SO_4_ and evaporated to a minimum volume (2.0 mL) for the analysis. The water elutes (fraction V) were liquid–liquid extracted with chloroform, dehydrated with anhydrous sodium sulfate and evaporated to a minimum volume (2.0 mL) for the analysis. The recovery of clomazone was determined by fortification of the soil samples at the concentration 0.01, 0.1, 1.0 mg·kg^−1^, and the water samples at the concentration 0.01, 0.1, 1.0 mg·mL^−1^ in three replicates. The average recovery of clomazone for the soil samples was 93 %, for the water samples 96 %. The quantification limit of the method for the soil samples was 0.0005 mg·kg^−1^, for the water samples was 0.00025 µg·mL^−1^. The standard solutions of clomazone at the concentrations from 0.0005 to 0.01 mg·mL^−1^ were prepared in chloroform.

Gas chromatography was used to determine the concentrations of clomazone. PerkinElmer Clarus 600 gas chromatograph was equipped with a MS detector and Elite 5MS column (30 m × 0.25 mm, 0.5 µm film thickness). The operating temperatures were: detector 320°C, oven temperature 100°C – 1 min, 10°C·min^−1^ to 220°C – 5 min, 25°C·min^−1^ to 300°C – 2 min. The carrier gas was helium with a flow rate of 1.0 mL·min^−1^. Under these conditions the retention time was 13.02 min. To determine clomazone in the samples, the electronic ionization method, type EI+, was used. Clomazone qualitative analysis was based on the mass spectrum and ions: 125, 204, 239 which are characteristic for this compound. Quantitative analysis was performed by the comparative method, based on the calibration curve (y = 80491x − 26.13; n = 6, R^2^ = 0.999).

All experimental data was calculated using statistical program Statistica 10.0 for Windows. Statistical analyses were performed using two-way analysis of variance (ANOVA) to determinate the formulation and the soil type effect on the mobility of clomazone in the soil. Means were compared by Tukey test and expressed as mean ± SD. The differences were considered to be significant at a significance level *p* = 0.05.

## Results and Discussion

**Fig. 1:**
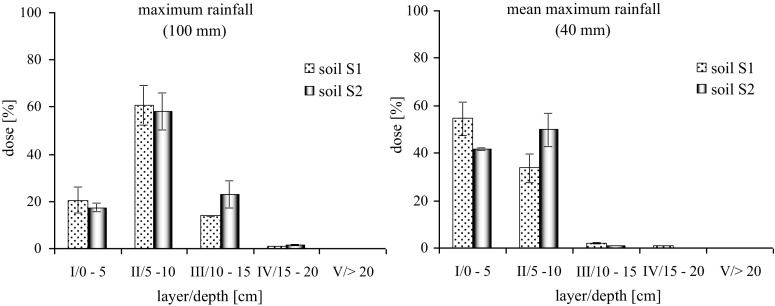
Mobility of clomazone as formulation EC in soil

**Fig. 2:**
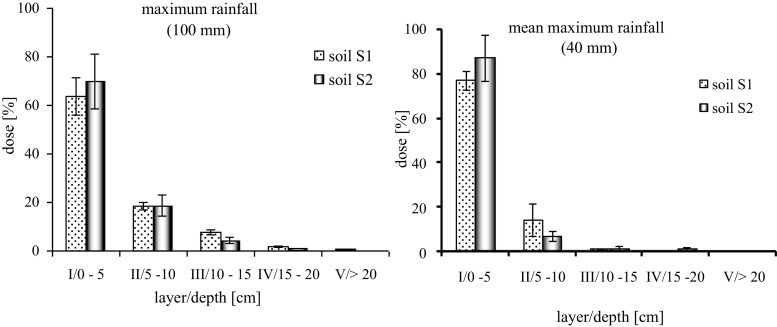
Mobility of clomazone immobilized in alginate matrix in soil

The results of the research indicate a significant relationship between the formulation and the mobility of clomazone in the soil. In the case of the emulsifiable concentrate formulation (Command EC), the irrigation with a water amount corresponding to the maximum rainfall (100 mm) resulted in a distribution of clomazone to each layer under the analysis. Similar relationship was found both for the loamy sand soil S1 as well as the sand soil S2 (Fig. 1). The highest concentration of clomazone was determined in the layer II at the depth of 5–10 cm: for S1 60.76 % ± 8.17 %, and for S2 58.32 % ± 7.87 % of the applied dose. In both soils, the significant amounts of clomazone were also found in the layers: I, III and IV. The concentration of clomazone determined in the layers V (leachate) were insignificant (<1 %). The statistical analysis conducted with the use of Tukey’s test (*p* = 0.05) reveal the significant difference in the concentration of clomazone, between S1 and S2 only in the layer III. Irrigation with the amount of water corresponding to the average maximum rainfall (40 mm) resulted in the translocation of the herbicide in the EC formulation in soil. In both soil types, the highest concentration of the active substance was determined in the first two layers at the depth of 0–10 cm. The variation in the content of clomazone in the layers I and II of the loamy sand soil (S1) was significant and amounted to 54.55 % ± 6.92 % (layer I) and 33.79 % ± 5.93 % (layer II). The translocation of clomazone in the sand (S2) was found to be significantly greater, and the highest concentration of the herbicide was determined for the layer II as a result of its translocation from the layer I. The content of the active substance in the layer I was 41.75 % ± 0.06 %, and in the layer II 49.85 % ± 7.08 %. However, the differences between the two layers of S2 were not statistically significant. In the case of the average maximum rainfall (40 mm), the significant concentration of clomazone was also determined in the layer III at the depth of 10 – 15 cm (S1 = 2.30 % ± 0.49 %, S2 = 1.03 % ± 0.12 %), whereas in the layer IV the concentration did not exceed 1 % of the applied dose. The variation in clomazone mobility as recorded in S1 and S2 soil types were found to be statistically significant, as was confirmed by the results of the Tukey’s test (*p* = 0.05). The amount of water corresponding to the average rainfall (3.7 mm) did not significantly affect the translocation of clomazone in the soil as applied in the form of the commercial preparation Command 480 EC, since the applied dose of water did not translocate below the first analysed layer of the soil in the column, 0–5 cm (data not shown on figure). The application of the formulation based on sodium alginate caused a significant decrease in clomazone mobility in the soil. Regardless of the applied dose of water, the highest concentration of clomazone, ranging from 63.50 % to 100 %, was determined in the layer I at the depth of 0–5 cm. In the case of the maximum rainfall, clomazone released from the alginate matrix (S1 = 27.30 % ± 0.83 %; S2 = 24.43 % ± 1.45 %) was distributed mainly to the layers II and III. Both, in the case of S1 as well as S2, concentration of clomazone was determined at the depth of 5–10 cm (II) where it amounted to approximately 18 %, and at the depth of 10–15 cm (III) where the concentration was 7.57 % in S1 and 4.36 % in S2. In the layer IV (15–20 cm) and V (leachate >20 cm), concentration of the analysed active substance was below 1 % (Fig. 2). In the case of the average maximum rainfall, clomazone released from the alginate matrix (S1 = 15.20 % ± 1.82 %, S2 = 8.35 % ± 0.95 %) translocated into the soil at the depth of 5 – 10 cm. Concentration of clomazone >1 % was not found below the layer II. For the maximum (100 mm) and the average maximum rainfall (40 mm) the differences in the migration of clomazone immobilized in the alginate matrix in the soils S1 and S2 were not statistical significant according to Tukey’s test at *p* = 0.05. As it was the case with EC formulation, the average rainfall had no effect on the mobility of clomazone, as the applied dose of water did not reach below the first analysed layer of the soil, 0–5 cm (data not shown on figure). On the basis of the results of the experiment, it was found that the differences in the mobility of clomazone applied in the form of Command 480 EC preparation and alginate capsules are significant which was additionally verified with the use of Tukey’s test at *p* = 0.05.

Pesticide leaching through the soil is a particularly important issue in a number of environmental and agronomic problems. Leaching is considered to be the main cause of groundwater contamination with pesticides which is largely determined by physiochemical properties of the active substances, soils (mechanical composition, organic matter, soil moisture, pH) and rainfall.

As can be concluded on the grounds of the results of the experiment, the formulation has a significant effect on the mobility of clomazone in soil. Additional factor which affects the process is the amount of precipitation (the dose of water applied). In the case of clomazone, the soil properties did not affect its mobility in a significant way. Clomazone in the form of EC formulation is more prone to leaching than when it is immobilized in the alginate matrix. Primarily, the fact can be attributed to the high solubility of clomazone in water (Table 1) and a relatively weak adsorption in soil (K_d_ = 0.47–5.30). The values of the leaching potential LP (Fernández-Pérez et al. 2011), calculated on the basis of half-life time (DT50 in soil 28–117 days) and partition coefficient values K_OC_ (150–300 mL·g^−1^) as specified in the literature on the subject (Antonious 2000; Lian-Fang et al. 2004), indicates that clomazone is a substance characterized by its high lechability properties (LP = 2.2–5.1).

1 $$LP = \log (DT50_{soil} ) \cdot (4 - \log (K_{OC} ))$$

Consequently, clomazone presents properties, which indicate a groundwater contamination potential. The results of the monitoring studies of aqueous environment by Caldas et al. (2010) additionally acknowledge this fact. Residues of clomazone in the range of 0.20–0.82 μg·L^−1^ were found in water collected from 70 % of analysed wells.

The formulation based on the alginate matrix, that was used in our studies significantly reduces clomazone mobility in the soil profile. The release rate of clomazone from capsules in the soil was slower than in water. Values T_50_ (a release time of 50 % of active substance from the matrix) for clomazone in water was: for hydrogel capsules 1.09–1.34 h, for dry capsules 2.30–2.31 h (Włodarczyk and Siwek 2013). In soil this process is regulated by the applied dose of water (a rainfall in the natural conditions) and the amount of the active substance released from matrix, distribution of which depends on the physiochemical properties of soil.

The additional factors affecting on the kinetics of clomazone release from the alginate matrix to soil, and thus its mobility, is the occlusion of the alginate matrix surface by the soil particles and the slower diffusion of the active substance from the matrix in soil. Moreover, the substances contained in the soil solution may delay the translocation of the active substances to the aqueous phase (Fernández-Pérez et al. 2000b). The same relationship was obtained by studying the impact of the formulation on the mobility of pendimethalin and metazachlor in the soil. Pendimethalin and metazachlor, as commercial formulations Panida 330 EC and Metazachlor 500 SC, have the ability to move in the soil. The use of herbicides immobilized in the alginate matrix, reduce this process (Włodarczyk 2014). Additional technology which affects the behavior of herbicides in the environment is an adjuvants technology. Numerous studies show that adjuvants strongly influence pesticide delivery, uptake, redistribution, persistence and the final biological performance (Krogh et al. 2003). Research indicates also that adjuvants can reduce leaching of herbicides in the soil (Kucharski and Sadowski 2011).

The results of the current study demonstrated the capacity of formulation based on the CR technology to control the delivery of pesticides with high water solubility, thereby reducing the amount of active ingredients which leach, and thus indicating its useful application to prevent pollution of water environment.
